# The pesticides use and the risk for head and neck cancer: A review of case-control studies 

**DOI:** 10.4317/medoral.23962

**Published:** 2020-07-23

**Authors:** Augusto César Leal da Silva Leonel, Roberta Ferreti Bonan, Mariana Bitu Ramos Pinto, Luiz Paulo Kowalski, Danyel Elias da Cruz Perez

**Affiliations:** 1 Universidade Federal de Pernambuco, School of Dentistry, Department of Clinical and Preventive Dentistry, Oral Pathology Section, Recife, Pernambuco, Brazil; 2 A.C. Camargo Cancer Center. Department of Head and Neck Surgery and Otorhinolaryngology, São Paulo, Brazil; 3 Department of Head and Neck Surgery, University of Sao Paulo Medical School, São Paulo, Brazil

## Abstract

**Background:**

Tobacco, alcohol consumption, and HPV infection are the most common risk factors for head and neck cancer (HNC). Despite of this, recent evidences are growing on the association between long-term exposure to pesticides and the risk of chronic diseases, including different types of cancer. The present review evaluated in current literature evidence of an association between exposure to pesticides and the occurrence of HNCs.

**Material and Methods:**

A literature search of the case-control studies was conducted in the PubMed, Web of science and Cochrane databases. Methodological quality of each study was rated with the Scottish Intercollegiate Guidelines Network (SIGN 50) checklist.

**Results:**

One thousand and thirty-five studies were identified and twelve met all criteria and, therefore, considered for quality assessment and data extraction. According to SIGN 50 criteria, six studies received an overall high-quality. All the studies considered of high quality found a positive association between exposure to pesticides and different HNC sites, including larynx, pharynx and nasal cavity. In addition, the increased risk was associated with the frequency of exposure.

**Conclusions:**

Finally, improving pesticide users' awareness of their risks and proper handling, as well as adopting protective measures such as the use of personal protective equipment, appear to be effective in reducing human health damage.

** Key words:**Case control studies, head and neck cancer, pesticides, review, risk factors.

## Introduction

Cancer is a global health problem. In 2018, it was estimated that 18,1 million new cases and 9,6 million cancer deaths occurred worldwide (1). Most head and neck cancers (HNC) arise at the upper aerodigestive system, being categorized based on the area of the head or neck in which they originate, including the lips, oral cavity, pharynx, larynx, nose, paranasal sinuses and nasal cavity. There are also thyroid and salivary gland tumors, and others less frequent (2). Lips and oral cavity are the second most common sites for HNC, with annual incidence of 354,864 cases (2.0% of all cancers) in 2018 (1).

The causes of the different types of cancer are multifactorial. However, alcohol consumption, cigarette smoking and HPV infection are the most common risk factors for HNC (2,3). Despite of this, evidences are growing on the association between long-term exposure to pesticides (in occupational or nonoccupational settings) and an elevated rate of chronic diseases, including different types of cancer (4,5). The exposure of humans to pesticides is possible through ingestion, inhalation or dermal routes (4). Studies conducted by the Agricultural Health Study have shown suggestive evidence of increased incidence of prostate, lung, colon, pancreatic, bladder cancer, besides leukemia and multiple myeloma, with increased lifetime exposure to certain pesticides (6-11).

According to the World Health Organization (WHO), pesticides are chemical compounds that are used to kill pests, including insects, rodents, fungi and unwanted plants (weeds). Pesticides are used in public health to kill vectors of disease, such as mosquitoes, and the use in agriculture is focused to kill pests that damage crops. In 2007, the amount of pesticides used in the world was approximately 2.4 million tons, totalizing a cost of more than U$35 billion (12).

The International Agency for Research on Cancer (IARC), an agency of the World Health Organization, publishes the monograph series on the Evaluation of Carcinogenic Risks to Humans, which is widely used to identify environmental carcinogens and to help guide government policy in protecting people from the risk of cancer due to dietary, environmental and occupational carcinogens. Currently, IARC classifies two pesticides as human carcinogens (Group 1) namely, the insecticides pentachlorophenol (non-Hodgkin lymphoma and multiple myeloma) and lindane (non-Hodgkin lymphoma) (13,14). The pesticides of group 2A were classified as probably carcinogenic to humans, because they presented limited evidence for carcinogenicity in humans. This group include herbicides glyphosate (non-Hodgkin lymphoma) and the insecticides diazinon (non-Hodgkin lymphoma, leukemia and lung cancer), melathion (nonHodgkin lymphoma and prostate cancer), 1,1,1-trichloro-2,2-bis (p-chlorophenyl)-ethane (DDT) (non-Hodgkin lymphoma, liver and testis cancers), dieldrin, and aldrin metabolized to dieldrin (breast cancer) (13,14). The insecticides trichlorophenol, tetrachlorvinphos and parathion showed sufficient evidence in animal carcinogenesis experiments, but the epidemiological data were considered inadequate. Thus, they were classified as possibly carcinogenic to humans (Group 2B) (13).

Most studies that investigate the role of pesticides and cancer risk have focused on cancers other than HNC. Recently our group conducted a review evaluating the association between pesticide exposure and the risk of head and neck cancers, considering cohort studies, and observed that the number of publications is small. Moreover, the literature did not support clear evidence for association between pesticides exposure and HNC (15). The aim of this review of case-control studies is to evaluate the possible association between exposure to pesticides and HNC.

## Material and Methods

- Search Strategy

Online databases PubMed, Cochrane and Web of Science were accessed on September 2019, using the following combined keywords: “pesticide”, “head and neck cancer”, “oral cancer”, “buccal cell”, “oral cavity cancer” “mouth cancer”, “lip cancer”, “larynx cancer”, “pharynx cancer”, “nose cancer”, “sinuses cancer”, “thyroid cancer”, “salivary glands cancer”, and “agriculture”.

- Study Eligibility criteria

Articles were included if they m*et al*l of the following criteria: 1) Case control study design published in the English language; 2) cases included at least one head and neck cancer sites, including oral cavity, lip, larynx, pharynx, nasal cavity, paranasal sinuses, thyroid, and salivary glands. Two independent investigators (ACLSL and RFB) reviewed titles and abstracts for relevance based on the inclusion criteria. Full-text evaluation was performed when articles could not be excluded from the first screening. In discrepancies between these 2 investigators, a third independent researcher (DECP) moderated the differences.

- Quality assessment and data extraction

Methodological quality of the included studies was independently assessed by two reviewers (ACLSL and RFB) using the Scottish Intercollegiate Guidelines Network (SIGN 50) checklist for Case control studies. For each study, the following items were evaluated: internal validity, selection of subjects, assessment methods, and confounding factors. Reviewed articles were classified as “high-quality” (majority of criteria met and very low risk of confounding or bias); “accepTable quality” (most criteria met, but with an increased risk associated of confounding or bias); and “modest quality” (accentuated risk of confounding or bias or significant flaws relating to critical aspects of study design).

- Data extraction 

Data were independently extracted from reports, by two members of the research team, according to sample characteristics (cases and controls, gender and age), study area, evaluation time, studied pesticides, risk assessment, HNC studied, considered confounders, and risk estimative.

## Results

**Figure 1:**
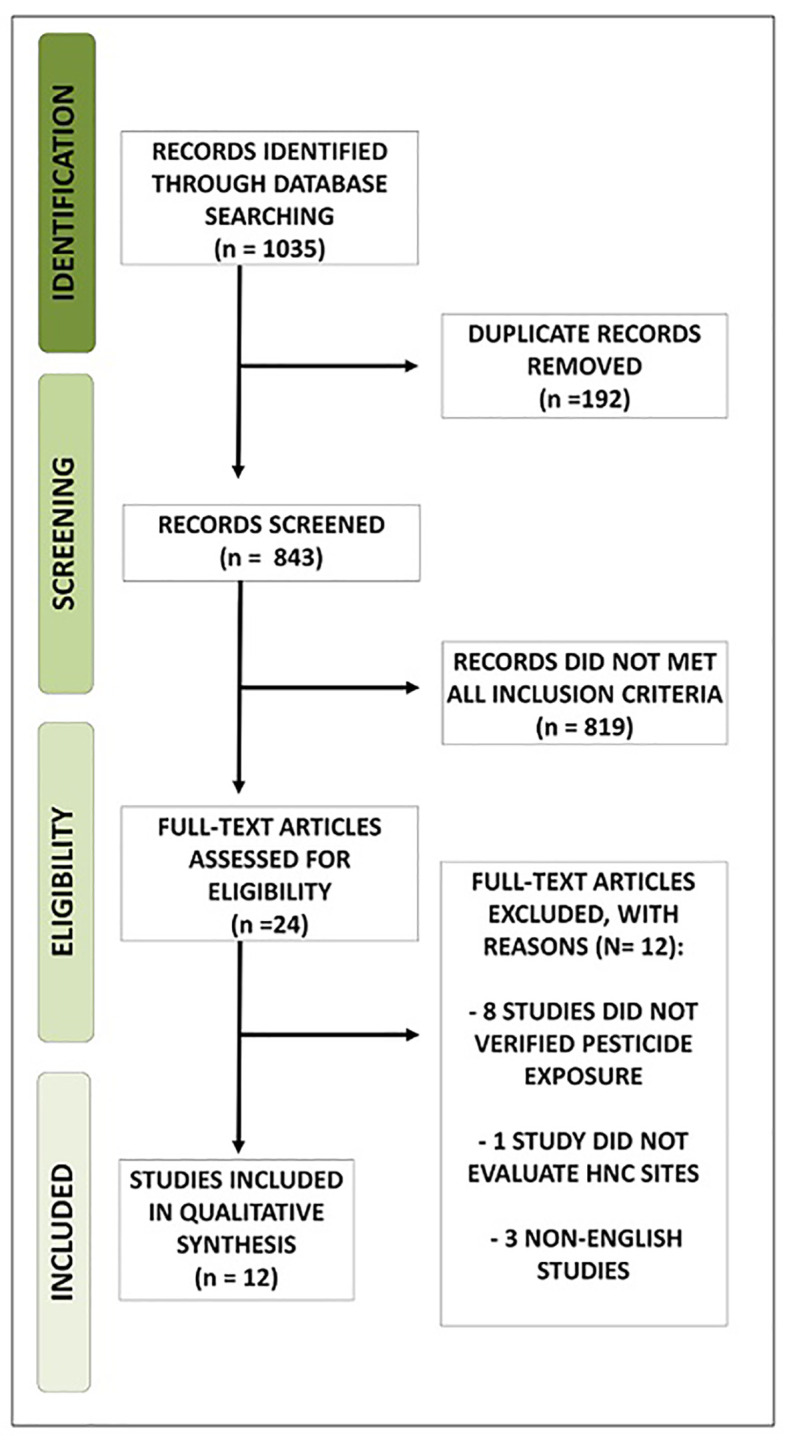
Study Flow Diagram.

The initial search resulted in 1035 studies. The articles found were published from 1962 to 2019. From the total number of citations provided, 192 duplicated studies were removed. All abstracts were hand searched regarding the eligibility criteria, and 24 abstracts were selected. After reading the full texts, another 12 articles were excluded because they did not directly describe pesticide exposure, did not verify the outcome at the selected cancer sites in the present study or were not written in English. Twelve studies m*et al*l criteria and were considered for quality assessment and data extraction (Fig. 1).

Among the selected studies, most were conducted in Europe (16-20), followed by North America (21-23) and Asia (2,24). Only one study was conducted in South America (25) and another one in Oceania (26). No studies were found in countries from Africa. Only one study focused exclusively on non-occupational exposure to pesticides (18). The verified cancer sites were pharynx (2,17,18,20,23), larynx (2,16,20,25), thyroid (2,8,22,26), nasal cavity and paranasal sinuses (2,18,21,24), nasopharynx (23), oral cavity, oropharynx, lip (2,20), and salivary glands (2). Most studies did not evaluated a specific type of pesticide (16-18,20,21,23-26). In all selected studies the risk assessment was evaluated through questionnaires or interviews.

Heterogeneous results were found among selected studies when the association between pesticide exposure and HNC cancer was evaluated. Some studies did not found evidence of any association between pesticides exposure and HNC (16,19,21,22,26). On the other hand, most of them found a positive association between pesticide exposure with cancer in larynx (2,20,25), pharynx (17), nasal cavity and nasopharynx (18), and sinonasal areas (22,24).

According to SIGN 50 criteria, 6 selected studies received an overall high-quality (2,17,18,20,24,25), 2 studies were considered with accepTable quality (22,23), and 4 studies were classified with a modest quality (16,19,21,26).

The high-quality studies had shown concern about reducing possible biases in the study, including selection and recall bias, with consideration of important confounders for HNC cancer, such as tobacco and alcohol, sociodemographic factors (age, sex), and personal and family health history, in addition to use of cases and control with comparable characteristics. The analysis of the frequency, timing and mode of application of pesticides and the use of personal protective equipment also make the study more reliable.

All the studies considered of high quality found a positive association between exposure to pesticides and HNC. Only one of them specified which pesticides were evaluated (2). General characteristics, including country of study, sample size, study population (Table 1), type of exposure and risk assessment (Table 2), as well as HNC sites studied, outcome, estimated risk, and considered confounders (Table 3) of high-quality selected articles are shown.

The Table 3 exhibits the main findings from high-quality studies, which showed a positive association between exposure to pesticides and increased risk for different HNC sites. In addition, the use of insecticides in private residences was also associated with increased risk of nasal cancer (18).

**Table 1:**
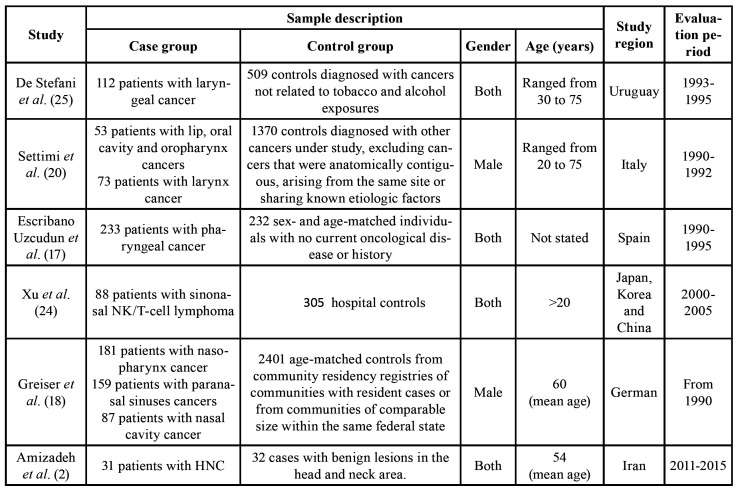
General characteristics of high-quality selected articles.

**Table 2:**
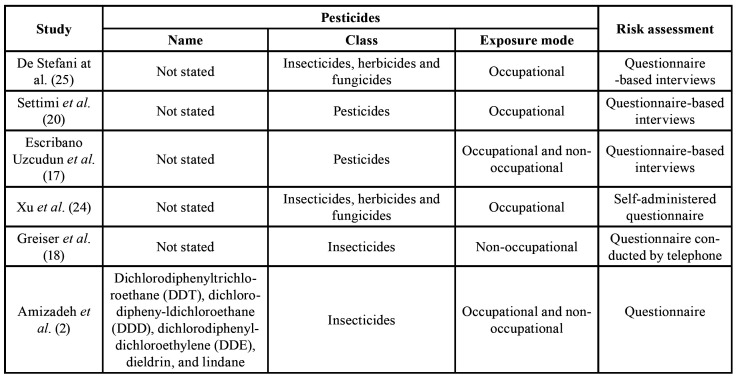
Type of exposure and risk assessment of high-quality selected articles.

**Table 3:**
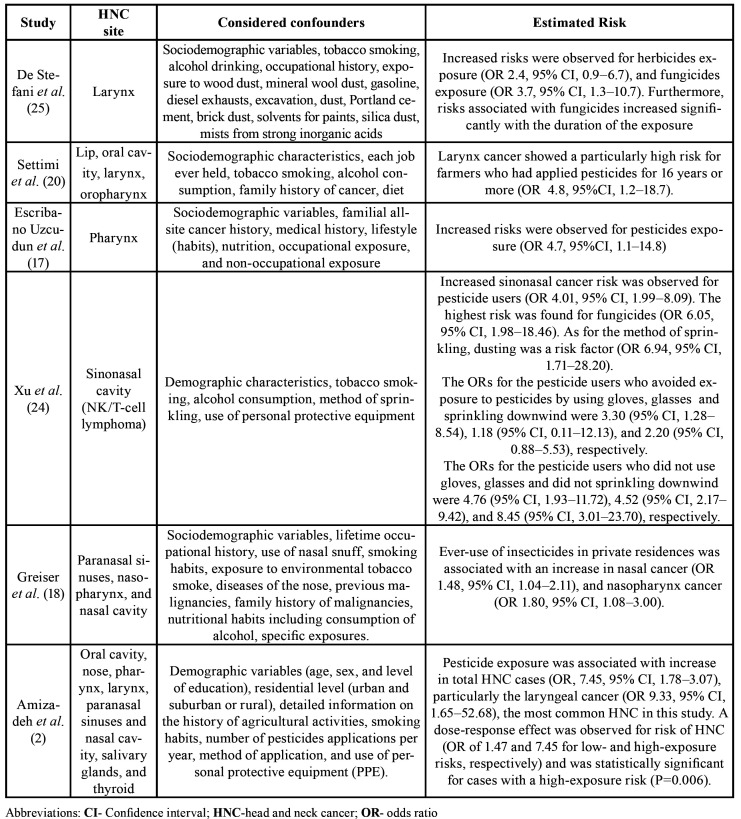
HNC sites studied, assessed outcome, considered confounders, and estimated risks.

## Discussion

The majority of head and neck cancers includes tumors that affect the upper aerodigestive system and have a multifactorial origin, which includes physical, chemical and biological factors (2). Although smoking, alcohol and HPV infection are known to be the most common risk factors for HNC, diet, lifestyle, family history of cancer and radiation exposure are other significant risk factors (17,22,27).

Pesticides are used for killing pest organisms and are related to various health problems in humans, including chronic impacts such as allergies, neurological and reproductive disorders, and cancer (2). In the present study, case-control studies were selected to access the possible relationship between exposure to pesticides and HNC. Observational studies, such as case-control and cohort studies, allow evaluating the causes or risk factors of diseases or health-related events. They represent important tools in situations where randomized controlled trials are not indicated, primarily for ethical reasons. Specifically, case-control studies are adequate to investigate outcomes with an extended latency period, in addition to being relatively fast and relatively inexpensive, with fewer subjects (28).

A limited number of case-control studies were found evaluating the relationship between pesticide exposure and HNC. This information corroborates previous studies, which state that although pesticide use has been associated with cancer, few studies have been conducted involving human populations, especially relating pesticides and HNC (2,20). The selected studies were conducted in diverse geographical regions, except in the African continent. However, previous studies have shown that the largest numbers of pesticide poisonings and deaths occur in developing countries, associated with insufficient knowledge about the risks associated with pesticide use and its correct handling (29-31).

Among the 12 selected studies, most of them focused exclusively on occupational exposure to pesticides, despite their widespread use in residences of the general population (8,9). This is probably related to the information that pesticides are primarily used in agricultural areas, mainly by individuals involved in agricultural practice. On the other hand, the domestic exposure is relevant when considering the stability of these compounds, which generally remain sTable in the environment, being able to contaminate water and food (2).

In the current review, the association between exposure to pesticides and HNCs was variable. Most of studies found a positive association between exposure to pesticides and one or more HNC sites. Initially, heterogeneous results could be explained by the sociodemographic differences of the analyzed populations, different periods and frequencies of exposure to the pesticides, besides the variation among the evaluated pesticides, despite the most of the studies do not present this information. However, when considering only high-quality studies, all found a positive association between exposure to pesticides and different HNC sites evaluated (2,17,18,20,24,25).

A positive association between larynx cancer and exposure to herbicides and fungicides was found and, despite mechanisms being unknown, the authors were able to associate this increased risk to the duration of exposure (25). Moreover, they observed a potential risk when the interaction between tobacco smoking and fungicides was analyzed, especially between heavy smokers (> 36 packs/year) and ever exposure to fungicides. Similar results were found by Settimi *et al*. (20), which evidenced an increase in the risk of laryngeal cancer especially among younger farmers and agricultural workers who had been involved in pesticide application for at least 16 years. Likewise, Amizadeh *et al*. (2) have found a positive association between pesticide exposure and HNC, especially larynx cancer, after controlling for confounders. The increase in risk was correlated with the higher level of exposure and lower level of education of the subjects exposed to pesticides.

High risk of pharyngeal cancer was observed in users of pesticides after adjustment for tobacco smoking and alcoholic beverage drinking (20). In the same way, Greiser *et al*. (18) associated ever-use of insecticides in private residences with an increase in nasal cavity cancer and nasopharynx cancer. However, the increase in risk was restricted to never-smoking men. Xu *et al*. (24) found a positive association between exposure to pesticides and nasal NK/T-cell lymphoma. This risk could be reduced with the use of protective equipment and a careful sprinkling of pesticides.

Among the selected high-quality studies, the strengths of the studies included histological confirmation of HNC, potential exposure information, case population and controls with comparable characteristics, besides considering essential confounders including sociodemographic characteristics, smoking and alcohol habits, personal medical history and family background. Some of them still considered important aspects such as the method of sprinkling and the use of personal protective equipment during the application of pesticides (2,24).

On the other hand, accepTable quality studies have as main limitations minimal numbers of cases (22,23), making validation and reliability of the studies difficult, besides making it impossible to carry out complementary analyzes, such as the role of the intensity of the exposure in the evaluated outcome (22). The limited number of cases may be explained by the relatively uncommon nature of some HNC sites, mainly associated with specific variables such as exposure to pesticides. This reduces the probability in to found some positive association, in addition to increasing the possibility of false associations (22). Finally, studies considered of modest quality presented failures in the verification of individual exposure (19,21,26,27), and in considering essential confounders for the evaluated outcome (19,21,26), reducing the validity and reliability of the presented results (16).

Recently, our group conducted a review addressing the same matter, however, considering only cohort studies (15). Differently from the present study, the literature did not support a positive association between pesticide exposure and HNC. A previous meta-analysis performed to assess whether farmers had elevated rates for several cancers, the authors showed that the results for most cancers were heterogeneous according to the study design. Positive associations were more often found in case control studies, compared to cohort studies, but it can be better explained by differences on sample size and quality of collected information than by study design (32). In this current review, a meta-analysis would be valuable. However, the studies evaluated HNC from different sites, resulting in high heterogeneity.

In general, the association between pesticide exposure and HNC appears to be complicated to be assessed. Studies have shown that due to the variable nature of the pesticides, not all of them are known to be mutagenic, teratogenic or carcinogenic, as well as the different frequencies and intensity of exposure, geographic region, crop type, season, exposure pathways, application method and use of individual protection equipment can also generate different degrees of impairment to human health. Additionally, the interaction with other important risk factors such as alcohol and tobacco consumption were also determinant for the development of HNC (2,20,24,25).

Despite of this, it seems that a better level of education improves users' perception on the risks of the pesticides, which can lead to a change of posture in the use of these compounds. The higher care in handling associated with the use of available protection mechanisms leads to the minimization of possible health problems (2,24). This finding seems to be useful in the development of public policies capable of assisting the exposed population, aiming at their greater protection and conscious use of pesticides.

## Conclusions

Among the high-quality studies, there was consensus on the positive cause-effect relationship between pesticides and HNC, such as laryngeal, pharyngeal and nasal cancers, especially when the frequency of exposure was considered. To improve the education level of individuals exposed to pesticides in relation to risks for cancer, including HNC, should be a priority of public health policies.
